# Exploring energy grid resilience: The impact of data, prosumer awareness, and action

**DOI:** 10.1016/j.patter.2021.100258

**Published:** 2021-06-11

**Authors:** Rolf Egert, Jörg Daubert, Stephen Marsh, Max Mühlhäuser

**Affiliations:** 1 Telecooperation, Technische Universität Darmstadt, Darmstadt, Hesse 64289, Germany; 2 Department of Mathematics and Computer Science, Phillipps Universität Marburg, Marburg, Hesse 35037, Germany; 3 Department of Computer Science and Business Informatics, Provadis School of International Management and Technology, Frankfurt (Main), Hesse 65929, Germany; 4 Faculty of Business and IT, Ontario Tech University, Oshawa, ON L1G 0C5, Canada

**Keywords:** DSML 3: Development/Pre-production: Data science output has been rolled out/validated across multiple domains/problems

## Abstract

The transition of energy grids toward future smart grids is challenging in every way: politically, economically, legally, and technically. While many aspects progress at a velocity unthinkable a generation ago, one aspect remained mostly dormant: human electricity consumers. The involvement of consumers thus far can be summarized by two questions: “Should I buy the eco-friendly appliance? Will solar pay off for me?” However, social and psychological aspects of consumers can profoundly contribute to resilient smart grids. This vision paper explores the role of active consumer-producers (prosumers) in the resilient operation of smart energy grids. We investigate how data can empower people to become more involved in energy grid operations, the potential of heightened awareness, mechanisms for incentives, and other tools for enhancing prosumer actions toward resilience. We further explore the potential benefits to people and system when people are active, aware participants in the goals and operation of the system.

## The bigger picture

Energy grids are highly data-driven socio-technical systems, which have historically been top-level managed. They are in transition because of moves to different sources of energy as well as different kinds of producers of energy. We discuss the importance and benefits of having active and aware people to participate in the behavior of these grids, and their potential contributions to overall system resilience. The key learning points are that (1) energy grids are in a transition period and may—in this interim stage—suffer from less overall stability; (2) the resilient operation of energy grids strongly depends on increasingly active behavior of prosumers—i.e., consumers that are capable of producing electricity locally; and (3) we need expedient strategies and strong data infrastructures to support these prosumers in taking on an increasingly important responsibility in the context of power grid operations. Toward this end, we explore strategies for encouraging people to participate in processes that may help the grid in its interim stage and elaborate on the roles of energy grid data and the energy-related knowledge or these prosumers for supporting these strategies. Finally, we detail potential futures for enabling resilient energy grids through resilient people.

## Teaser

Energy grids are large-scale socio-technical systems that are in a transition process to move from centralized and fossil-fueled electricity production to an increasingly decentralized system supported by renewable energy sources. The paper discusses the importance of active and aware people in increasingly decentralized energy grids and explores strategies for encouraging them to take on more responsible roles in the context of energy grid operation.

## Introduction

Electricity is the boon of our modern age, yet the way in which it is delivered to consumers has often been something of a black box affair, in which the producers—large conglomerates and governments—benignly finance and operate massive and complex generation and distribution systems for the benefit of all concerned. One flicks a switch and, as if it were by magic, the light goes on. If it does not, the usual litany of “bulb out,” “did you pay the bill?” or “are the lights on next door?” arises. The point is that the people flicking the switch are, willingly or unwillingly, largely unaware of the feats which are accomplished to keep the power flowing hour by hour and incident by incident. They are, in other words, passive.

Recent advances, driven by various factors, have begun to lead us away from conglomerate to distributed power systems. Electric vehicles (EVs) not only roam the roads but are capable of powering homes. Solar panels are increasingly in evidence, wind and wave power likewise more popular. While renewable energy and large batteries on wheels have obvious benefits to the layperson (clean air is nice), they bring their own share of issues: the sun is not always shining, wind is not perhaps as predictable as atoms, and, heretofore hidden from the passive consumer, the ways in which loads are balanced and electricity delivered is less straightforward as a result. On top of this, the consumer becomes, in some instances, a *prosumer*, a consumer, *and* a producer of energy: shared from EVs, from solar panels on the roof, and so on.

What this means, and what it needs, is a more aware, *active* set of consumers, more information from between traditional producers (and infrastructure owners) and both prosumers and consumers, and more acceptance on the part of all that (1) we are in a transition period in which the traditionally black box yet overwhelmingly stable grid is being slowly replaced by less stable, perhaps more mercurial means of production which may require local management and, in the interim, perhaps less overall stability; (2) the stability is a function of how previously passive consumers behave in future; and (3) notions of resilience, instead of stability, are needed to help mitigate and manage what is to come.

Much of this is understood at a technical level, but energy production and distribution is no longer a technical system (if it really ever was). There is a need to appreciate the “socio-” parts of the socio-technical system that in fact exists. This means that there is a host of non-technical challenges that have to be understood and addressed for our brave new world to be resilient and beneficial to the humans in it. This includes aspects of education, awareness, activism, and incentivization for all concerned.

This paper explores some of these socio-technical challenges, why they matter, and some approaches to dealing with them based on what is currently known and done. While not prescriptive, it suggests programs that may be of help in our interim stage, and explores the role of energy grid data for supporting these programs. Finally, the paper elaborates on potential futures with an eye to enabling resilient energy grids through resilient people.

## Involvement of people in resilient grid operations

**Figure 1:**
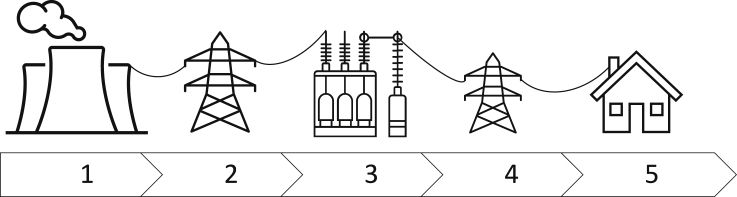
Sketch of the hierarchical electricity transportation

The mode of operation of the energy grid is based on a centralized, top-down producer to consumer electricity production and delivery process. In this process, electricity is produced by (1) large centrally located and (mainly) fossil-/nuclear-fueled producers (e.g., coal and nuclear power plants). The production is adjusted depending on currently available information from the grid (e.g., current demand) and forecast information (e.g., day-ahead demand predictions). These producers are mostly placed in remote areas. For transporting the generated electricity to the consumers, they produce electricity of high-voltage, which can then be (2) transmitted via transmission lines over long distances. Once the transported electricity reaches the vicinity of consumers (e.g., cities, industrial areas), (3) transformers are used to step-down the voltage level of the electricity to make it usable for the equipment of the consumers. Note that this stepping-down process may occur multiple times, transforming the voltage level depending on the requirements of the consumers located in the vicinity of the transformer (industrial appliances may require different voltage levels compared with residential appliances). Finally, (4) distribution lines transport the electricity to the (5) consumers, where it is used to supply the demand of devices. This process is visualized in a simplified example as Figure 1.

The involvement of people in the resilient operation of the described supply chain of the energy grid is ubiquitous. The various processes for controlling and monitoring electricity production are not fully automated and require manual interaction. Therefore, corresponding expert personnel is required, which is responsible for the correct operation of these processes. Furthermore, a variety of people are involved within the different transportation and transformation processes of the supply chain. Among those are, for instance, people responsible for maintaining equipment, such as transmission lines and transformers; personnel for monitoring and distributing electricity along different sections of the supply chain; and entities for providing ancillary services, such as offering balancing energy for frequency stabilization (e.g., operators of medium-sized hydro-electric power plants). People involved in the aforementioned processes are considered to take an *active* role in supporting the resilient operation of the grid. Consequently, these people are an essential part of the resilience of the overall system.

The second main group of participants in the grid is consumers (5), which represent the final stage of the depicted electricity supply chain. The role of the consumer can be further divided into large-scale consumers (e.g., large organizations or enterprises) and small-scale consumers, such as households. The focus of this work lies on small-scale consumers. However, the majority (if not all) of the aspects discussed in this work can be mapped to industrial consumers with minimal adjustments. In contrast to the people involved actively in production and operational processes, the role of the consumers is mainly a *passive* one. We define passive involvement of consumers as follows:

• *Necessity of involvement.* Consumers are required for maintaining an operating electricity grid. For electricity to be able to flow, demand needs to be present, which is then met by producing a suitable amount of electricity.
• *No/limited problem awareness.* Consumers mainly participate in the grid by freely consuming electricity as they need to. Here, the majority of the consumers are not restricted in their energy consumption (exceptions may exist for industry and large-scale consumers), as the local grid was designed in such a way that the operational limits of the infrastructure can handle peak-consumption situations depending on the majority of participants in the area (e.g., operational limits differ if the majority of consumers is industry compared with households). Simultaneously, consumers do not have any awareness of the consequences of their consumption behavior. For instance, they do not know if their current consumption is beneficial for the grid if the local production is currently high, or if their behavior challenges the resilient operation of the grid. In fact, for the majority of people electricity is mainly invisible.^1^ It only becomes more tangible if severe situations occur that can be directly perceived as they affect the use of electricity, e.g., blackouts. However, perceiving these severe consequences is not considered to be *awareness* with regard to their handling of electricity and corresponding impact.
• *No/limited responsibility.* Consumers define the demand through their daily use of electricity, without the need to be aware of the corresponding impact (exceptions may exist for certain industrial consumers). Since the grid, in the past, has been mainly supplied by large-scale central producers, controlling these individual entities was a much simpler task than controlling or restricting the consumption behavior of a large number of consumers. As a consequence, the active and responsible role for matching demand and supply was assigned to the producers in the grid, and consumers—up until now—did not take on a role that has a responsibility for maintaining resilient grid operation.

The amount of electricity that is consumed by these small-scale consumers, however, represents a non-negligible amount that can strongly impact the resilient operation of the grid. In 2017, the electricity consumed by small-scale residential consumers represented 27% of the overall final energy consumption in Europe.^2^ To improve the management of this amount of electricity and alleviate the task of maintaining resilient grid operation, different types of control schemes exist. These schemes aim at providing consumers with the ability to contribute to the resilience operation of the grid. The most prominent options are:

• *Incentive-based control schemes.* These schemes aim at incentivizing consumers to make adjustments in their electricity consumption behavior. Here, the main incentive is the reduction of the overall electricity bill of the consumers. The most prominent examples for such approaches are *real-time pricing*, *time-of-use rates*, and *critical peak pricing*, where consumers can save money by adjusting the points in time when they consume electricity.^3^
• *Direct load-control schemes.* These approaches aim to establish a contract between consumers and energy providers, which allows control entities to remotely adjust the consumption behavior of consumers. For this, certain devices are configured in a way such that they can be deactivated remotely to reduce the demand in the grid. For the current grid, the most suitable candidates for this scheme are large-scale consumers, such as industry.^4^ However, residential approaches are also being investigated.^5^

By accepting and adhering to any of these schemes, consumers can have an increased impact on the resilience of an energy grid. However, the involvement of the consumers within the resilient operation of the grid remains passive. Incentive-based approaches use the desire of consumers to reduce their *own* electricity bill to generate beneficial effects, such as reduced demand peaks. Load-control schemes represent a more direct approach, where a consumer grants an authority the capability for making adjustments, or act on demand of the authority without being aware of the problem situation or the impact generated by the action. However, such schemes are perceived as highly invasive as they intrude on the private life of people and may require changes in the behavior of participants.^1^ This may have a strong negative effect on the satisfaction of people participating in the aforementioned schemes. For instance, devices might not be available when they are required since they are deactivated remotely, or their operation is postponed. Moreover, not all schemes might be applicable in all countries, as regulations vary. For instance, energy pricing in the US can be based on variable pricing schemes, but Germany mainly uses constant pricing schemes.

The next section takes a closer look the factors that lead to the limited awareness and involvement of consumers.

## Lack of knowledge and motivation for active participation

Resilient energy grid operation is a topic that mainly concerns a very limited number of expert personnel responsible for operating and managing the grid. However, most people have a rather limited understanding of the need for resilience within the energy domain.^6^^,^^7^ Various aspects are (partially) responsible for this lack of understanding, but we emphasize two aspects we consider as main contributors. The first aspect is the high degree of *availability* of the grid, and the second one the *passivity* of the consumers related to resilient grid operation. The two aspects are explained in more detail below:

• *Availability.* From the perspective of the majority of consumers, the grid provides a high degree of availability. This is represented by the grid successfully providing a continuous supply of electricity. In current grids, this stable operation is achieved by nuclear- and fossil-fueled production, high-quality forecast mechanisms, and hot-standby resources for problem mitigation. In addition, experts can draw from extensive experience in consumer behavior, which supports the decision making for adjusting the production of centralized producers to meet the expected demand (e.g., regions vary in peak consumption times due to climate conditions). If problems occur, component redundancy (e.g., *N-1* reliability^8^) ensures stable operation despite failing components, and demand and supply mismatches are mitigated using standby resources or load-shedding mechanisms. Consequently, these mechanisms maintain the continuous operation of the grid as a whole, where no or only very few parts experience service deterioration. In cases where people experienced disturbances, these were mainly caused by incidents that were not attributable to individual consumers but were caused by external influences (e.g., strong meteorological effects damaging connection lines and separating areas within the grid).^9^ Consequently, this absence of major losses or reductions in service quality can lead to a deceptive feeling of security of supply.^10^ Considering this, there has been no need for consumers to understand domain-specific details about the resilient operation of the grid.
• *Passivity.* As explained in the section entitled “involvement of people in resilient grid operations,” the grid is operating as a demand-driven and centrally controlled infrastructure. A limited number of adjustable large-scale producers is responsible for supplying a large number of consumers that cannot directly be controlled and may strongly vary in their electricity consumption behavior. As a result, resilient grid operation was mainly concerned with the grid’s capability to absorb changes in the demand of the consumers. In contrast, the role of consumers is to provide demand, which is necessary for electricity to be able to flow, but does not actively contribute to the resilience of the energy grid. Moreover, the consumption of electricity is mainly invisible to consumers since they are used to a ubiquitous availability of power.^11^ As consumers do not have a role that is associated with active responsibility, there was no need for consumers to have knowledge about the impact of their actions, nor was there a need to acquire a profound understanding of the processes for resilient grid operation.

The transition to distributed smart grids (SGs) provides opportunities to reduce consumer passivity and turns consumers into prosumers—predominantly consumers in suburban areas with the capability to enter the solar power and EV markets. That leaves everyone else and the growing threats to the availability of the energy grid. Hence, to leapfrog power outages, we discuss the benefits of increased consumer involvement and explore corresponding methods in the following section.

## Increasing the involvement of people in resilient grid operation

Involving people to support the resilient operation of energy grids is becoming increasingly important but is accompanied by numerous challenges. In this section we begin with a discussion of the importance of data in involving people. Furthermore, the importance of the energy-related knowledge of people is highlighted. Finally, various methods for motivating people and increasing their involvement are presented.

### Data as a prerequisite for involving people

We previously emphasized that the majority of participants in energy grids are willingly or unwillingly unaware of the feats that need to be accomplished for maintaining a continuous supply of electricity during both mundane operation and emergency situations. Simultaneously, the behavior of these participants can strongly affect the resilient operation of the grid (see the section entitled “involvement of people in resilient grid operations”). In this work, we want to emphasize two crucial requirements for enabling a more active set of participants in SGs: firstly, participants need to be able to assess various information that can support them in conducting active decisions toward supporting the resilient operation of the grid; secondly, participants need to be able to understand provided information to derive actions that either support their own interests or the overall resilience of the grid. Various information can be made available as data in SGs but we distinguish between the following three categories:

• *Grid data.* General information about the (potentially autonomous) SG, such as grid frequency, current power flow, and available reserve capacities. Such information may support participants that possess sufficient knowledge to interpret this information to make informed decisions about adjusting their local behavior. However, the representation of technical terms and units, such as kilowatts, limits the understanding of laypersons.^1^
• *Local data.* Information about local energy-related parameters, such as production, consumption, environmental information, and the state-of-charge of batteries. Similar to the grid data, knowledge of the participants is required for interpreting this information and for deriving corresponding actions.
• *Enriched data.* As enriched data, we consider a processed and extended type of data that may encompass combined information from the previous categories. This category of data should be provided to a participant in such a way that it aids the understanding of the situation of the grid, the local situation, and may allow deriving actions for the participants more easily. For instance, among such enriched data can be time-series information of how local energy saving impacted the operation of the SG (e.g., electricity saved over the course of the last 5 days could be used to power a certain number of important loads). These data can be presented to the user in meaningful and simple ways, e.g., via in-house displays.^12^ Such enriched data simplify the process of understanding the impact of local actions and may support decision making toward future actions.

Since SGs are envisioned to be strongly coupled with a large information and communication technology (ICT) infrastructure, and the digitization of cities as a whole is likewise progressing, it can be assumed that data can be made available on a large scale.^13^ This development is further supported by the ongoing digitization of homes (e.g., smart homes) and enterprises, which allows receiving of data and supports bidirectional communication.^14^ Consequently, SGs provide the necessary technical infrastructure and resources to generate, disseminate, and present the aforementioned data to participants in the grid.

However, technical solutions, such as the simple presentation of data, are insufficient to generate a more active and involved set of participants in energy grids.^12^ A variety of social aspects need to be addressed, which may impede the acceptance of technical advancements if neglected. For instance, the development of data standards and the mitigation of the privacy concerns of participants can improve the trust of participants in the system.^15^^,^^16^ Furthermore, depending on the category of data, varying degrees of energy-related knowledge are required to understand provided data and derive suitable actions. In the following, we motivate the necessity for increasing the energy-related knowledge of participants in SGs.

### Increasing energy-related knowledge

We begin with the assumption, to paraphrase Bacon and Hobbes,^17^ that knowledge about power is empowering. Therefore, increasing the energy-related knowledge of people aims at enabling them to understand the different categories of data provided by the energy grid and make informed decisions to support its resilient operation. To that end, our goal is to help consumers and prosumers understand more about how the system they are using produces, distributes, maintains, and restores power in different circumstances (and with differing tools and sources). Moreover, people should become more aware of the fact that they can—and should—contribute to the stable operation of the grid, especially during the transitioning process toward SGs and in emergency situations.^18^

Extending the energy-related knowledge of people is no mean feat, since there is a great deal that is assumed or taken for granted at present. It is also unlikely to be a speedy process: some of the information is technical, some is irrelevant to different kinds of consumers, and so on, and learning is a curiosity-driven process, not one that can be forced on consumers. Nonetheless, to use SG data efficiently for the benefit of the individual or the grid as a whole, increasing the corresponding knowledge is crucial.

There are several different ways in which utilities are beginning the process of educating and informing their customers, however. For instance, Hydro One in Ontario uses an App- (and online-) driven approach to allow consumers to identify when power outages occur, as well as examine a map of current outages with estimated times to fix. Text messages are also used for outage alerts and potential environmental hazards (thunderstorms, for instance).^19^ The intent is to inform the customer (and perhaps reduce calls to information/help centers!), but the effect may well be that the informed customer is more able to cope with (and prepare for) outages and problems. Indeed, a continual process of awareness for the informed *citizen* is required. In this process, education and awareness of infrastructure is of itself a worthwhile act. We can conjecture that, if there is an awareness process from youth to adulthood, the result will be better able citizens who are part of a resilient system and capable of contributing to make it work better.

Knowledge and awareness, while laudable goals, are likely not the only answer to helping infrastructures remain operationally resilient, however. There may well be limits to what people want to or can learn, for instance. Also, in an interim phase, getting valid knowledge and status information to customers to help them make better decisions is likely to be more problematic than in the future when distributed power systems are more widely available, strongly integrated with ICT and commonly understood. Furthermore, the simple presentation of information to people is insufficient to increase energy awareness and actionism.^1^^,^^12^

Other techniques that do not heavily rely on profound domain knowledge may be viable for encouraging people to become more actively involved, particularly during the transitioning period toward SGs. In this context, *nudging* represents a prominent technique for affecting the behavior of people. *Nudges* represent small “adjustments” to the presentation of choices people have in a specific context, without limiting these choices.^20^ The goal of these adjustments is to encourage people to make certain preferred choices by targeting automatic cognitive processes. These preferred choices should then yield a benefit for the entity presenting the choices and/or the people making them. For instance, nudging has been successfully applied in both physical and cyber contexts, by encouraging people to make healthy food choices, or by guiding them to generate stronger passwords.^21^^,^^22^ Furthermore, nudging was also successfully applied to encourage people to reduce their overall electricity consumption.^23^

However, while using nudging techniques may reduce the amount of domain knowledge required, other challenges remain. For instance, local ICT and *enriched data* are required to present choices and nudge people toward the ones that are beneficial for the current situation of the individual or the energy grid as a whole. Furthermore, aside from the technical implementation of such a system, people need to be willing to support such a concept in the first place and, once it is in place, remain motivated to participate. Related studies also show that knowledge and awareness are among the most impactful factors affecting the peoples’ willingness to become increasingly active within the energy domain.^24^ However, aside from increasing the domain knowledge, additional measures can be used to incentivize people to become and remain increasingly active.

### Motivating people to participate in resilient grid operations

Involving people in processes of improving resilient grid operation requires the acceptance of these consumers/prosumers first. Afterward, they need to remain motivated to participate continuously. Motivation is a complex topic that gained a lot of attention in the scientific community for many years.^25^ It is a crucial prerequisite, as participating in the aforementioned processes may cause disadvantages for the individual at first glance: financial investments into technological foundations, such as smart energy meters, may be necessary to participate. Such investments into novel technologies that improve the ecological handling of energy depend on a variety of different factors, such as the financial status of the prosumers, their personality, and their social network.^26^^,^^27^ Other types of investment are preparations that allow people to fulfill their basic needs during emergency situations. Such preparations can be emergency rations, e.g., purchasing long-lasting food reserves and maintaining auxiliary gadgets, such as battery-powered radios. Many of these investments are of a preemptive nature and may never be used before they expire. While this can be interpreted as a positive event, since the absence of an emergency situation means that the grid remained operational, people are reluctant to spend money on maintaining such preparations. A large study in Germany showed that only 70% of the people have sufficient food and water supplies to support themselves for up to 4 days. However, the German government recommends 14 days as a baseline for how long people should be capable of supplying themselves.^28^

Aside from cost aspects, potentially negative effects caused by actions that become necessary for maintaining the operation of the grid can diminish the motivation of people. Similar to the consequences of direct load shedding, where partial blackouts are experienced by a few consumers, actions that result in a conflict between the expectations of people and the current situation, diminishes the overall satisfaction of people. For instance, the state-of-charge of EVs may deviate from the state expected by the user, if it was used for grid stabilization purposes. If a person decides to use the car in this situation, they may not be able to travel the expected distance with the car. This can affect the daily life of people severely and decreases their satisfaction with participating in a system that leads to such conflicts. Moreover, the impact of supportive actions on the motivation of people cannot solely be defined by individual behavior changes, but may depend on numerous aspects, such as other people in the house and the peculiarities of the current situation of the person experiencing the conflict.^1^^,^^29^

Consequently, aside from investigating technical solutions to minimize the impacts on user satisfaction, it is crucial to support the motivation of users and to encourage them to participate in novel concepts to improve grid resilience despite emerging drawbacks.^30^ Various techniques can be applied to counteract the impact of emerging drawbacks on the motivation of people. In the following, several of these techniques are introduced.

#### Reimbursements

Reimbursements are a form of extrinsic rewards whose potential effects on motivating people are strongly discussed by experts.^31^^,^^32^ Nonetheless, these systems are used ubiquitously in our modern society (e.g., salary).

The most common way of reimbursing people for participating in measures to improve grid resilience is based on monetary benefits. For instance, prominent programs aim at reducing peak loads of electricity by offering to reduce people’s electricity bills as an incentive to avoid or reschedule using appliances at specific peak times.^3^ This represents a more indirect approach, where the reward is provided implicitly to the user as they are required to *pay less* instead of receiving money for their actions.^33^ However, the success of these approaches is ambiguous. On the one hand, dynamic pricing schemes are capable of achieving (significant) reductions in energy consumption and save money in the process; on the other hand, their success in the current grid is rather limited.^34^^,^^35^ Nonetheless, (monetary) incentives will be an important means to motivate people to participate in future resilient grid operations.^1^

With the increasing distribution of energy resources, people become less dependent on a single energy provider. Therefore, alternative market designs can change the landscape of energy trading.^36^ In scenarios such as peer-to-peer trading, direct reimbursement strategies become viable. Another reimbursement approach that is closely related to using money as an incentive is based on so-called customer loyalty programs.^37^ These programs are commonly used within various enterprises to strengthen relationships with their customers, with the ultimate goal to encourage them to buy additional products or use the services of the company. Among the most prominent programs are frequent flyer programs of airlines, where customers will receive bonus miles for each mile they travel with the corresponding airline. These miles can then be used by the customer as a currency to purchase goods, bonuses, or services from the airline (e.g., class upgrades within flights with the airline). The impact of customer loyalty programs has been found to improve the various relation aspects between customers and energy providers, strongly depending on the type of the provided reward.^38^

Various types of data are relevant for such reimbursement-based motivation approaches and may also require different degrees of knowledge about the energy grid. For instance, raw grid data about the current electricity price or the current stress on the energy grid (e.g., the traffic light concept of the German Association of Energy and Water Industries^39^) require the consumer to understand how different actions may affect their electricity bill and the energy grid. Furthermore, the simple display of information is insufficient to motivate people to engage with the energy grid for a longer period of time, but it can be used to support the process.^33^ In contrast, using enriched data or nudging approaches based on local information may build upon the simple display of information and alleviate the process of conducting actions by highlighting the benefits or drawbacks of different decisions.^23^

#### Social rewards

In contrast to extrinsic rewards, social rewards aim at affecting the motivation of people by means that result in the feeling of being rewarded.^40^ The longing of people to perceive this reward should then increase their motivation for taking actions that support the resilient operation of the grid or are beneficial for themselves. We distinguish between three categories of social rewards. The first is based on the individual’s *morals and values*. The second one uses people’s *sense of achievement*. The third is based on the *sense of comparison*. The actual *gain* from these categories, which is perceived as a reward after or while participating in resilient grid operations, is based on the personal feelings of the individual. Here, a gain can be represented as an improvement or intensification of positive feelings (e.g., increased happiness, intensified feeling of doing something *good*), or the dilution of negative feelings (e.g., reduced feeling of guilt toward something). The different categories and how data can contribute within these categories are described below:

• *Morals and values.* This category represents the concept of people being rewarded for acting *well* according to the standards of the individual’s moral and intrinsic values. In particular, the reward is derived from the alignment of the task with the individual’s perception of morals and values in the context of the task.^41^ For instance, an individual that considers the transition of energy grids toward SGs important may feel increasingly good by purchasing renewable energy sources (RESs), such as a solar panel, since this action is considered valuable for the transitioning process. The reward for this action can be derived without the need for any external incentives. In addition, the rewards of this category do not require other individuals in the process. Different types of data can support the process of feeling rewarded by providing additional information about the impact of the conducted action. For the solar panel example above, such information could be the amount of electricity produced locally or the amount of excess electricity that was transferred into the grid over a certain period, which supports the energy grid stability.
• *Sense of achievement.* The feeling that is perceived by achieving previously set goals is closely connected to the feeling that is derived from the task of collecting, where people are intrinsically motivated to possess various similar objects of interest.^42^ This category relates to the concept of feeling rewarded for accomplishing certain goals that can be associated with the conducted actions. Another closely related concept that uses accomplishing goals and earning achievements for completing tasks is the field of gamification.^43^ For energy grids, conducted actions should ideally improve the situation of the individual and the resilient operation of the grid. Corresponding goals can be set both intrinsically (e.g., set based on local information or intrinsic personal goals) and externally (e.g., city-wide efforts to decrease the carbon-dioxide footprint). Once such goals are achieved, the feeling of reward can be derived from the individual’s perception of accomplishment. Various data can support techniques that use the sense of achievement to induce a feeling of being rewarded. For instance, local and enriched data can be used to indicate how strongly an individual contributes to extrinsic goals (e.g., provide a measure for indicating the local reduction of CO_2_ emissions), or the progress toward intrinsic goals (e.g., the reduction of the average electricity bill compared with the average of the previous year).
• *Sense of comparison.* The tendency of people to compare themselves to others is an established concept for self-evaluation and self-adjustment in a social context.^44^ One major goal that is supported by such a comparison is to verify the view of oneself and a corresponding tendency to maintain a positive feeling about the self. To protect this positive self evaluation from negative feedback, it is likely that people are willing to engage in a variety of mechanisms. In the context of resilient grid operations, this enables the development and integration of different mechanisms using the concept of comparison as a means for motivating people. Various data and the ICT infrastructure of SGs can be leveraged to inform people about the actual impact resulting from their participation in supportive mechanisms. For instance, people may receive notifications about the number of appliances in the grid that could be supplied with the amount of electricity they saved by their economic handling of energy. This information may boost the self esteem of the participant, resulting in a positive self evaluation and increasing the motivation for further participation.

Another aspect in the context of self-evaluation is the concept of competition, where people can evaluate themselves by comparison with others. Data can support people in comparing their own actions and the achieved impact with others, such as their ecological performance for managing their local energy consumption.^45^ Toward this end, mechanisms can use local and enriched data to inform people about their performance and impact compared with other comparable peers (e.g., equally consuming households in the neighborhood, city, or on a larger scale). The demonstration of people’s performance can then be used for self-evaluation and can motivate them to improve their ecological management of electricity based on their desire to win, or at least to do better compared with others.

#### Legal compliance

The motivation generated by complying with legal requirements is not generally derived from the feeling of being rewarded, but from the feeling of successfully avoiding repercussions. Nonetheless, the strategy of establishing official laws and regulations to “encourage” people to act—or at least to not resist—has already been conducted within the energy domain. A prominent example of such a case is the implementation of the Smart Meter Operation Act as a part of the Digitisation of the Energy Turnaround Act, which was adopted in Germany in 2016.^46^ This Act specifies, among other aspects, that the installation of smart meters at a specific location is mandatory if the total annual energy consumption of such a location exceeds 6,000 kWh or the locally installed production capacity is more than 7 kW. Consequently, people with correspondingly high consumption and production capacities needed to install smart meters to comply with the legal requirements.

The use of data in this context to support people’s ability to avoid repercussions depends heavily on the legislation and corresponding circumstances. An example could be—assuming an emergency situation in the energy grid—the limitation of electricity consumption per consumer during a specific time period. Such situations can appear if power line capacity is insufficient for distributing the required amount of electricity. Similarly, if an SG operates autonomously from the main grid as a consequence of a major problem in the grid, available production capacity may be severely limited and based on local RESs and batteries. In such a scenario, laws could be applied that define specific consumption and production behaviors to ensure a continuous operation of the SG until the problem can be resolved. Various data can support both consumers and producers in adhering to such laws, for instance, by presenting relevant information if the current behavior achieves adherence, or which adjustments are required to do so.

## Active prosumers in future energy grids

Consumers are in the process of evolving toward prosumers—capable of both consuming and producing electricity. Simultaneously, the advancements of ICT infrastructure, smart homes, and the availability of increasing amounts of data enable consumers and prosumers to take on a more active role within SGs compared with their mainly passive involvement in current energy grids. Understanding the challenges and opportunities that accompany this change of role can have a strong impact on the resilience of SGs. In the following, areas of influence are discussed, where increased energy-related knowledge and growing motivation for the participation of the individual may yield beneficial effects for resilient grid operation.

### Understanding the necessity for changes in the energy grid

The energy grid is already undergoing changes in both structure (e.g., increasing introduction of numerous distributed RESs) and control concepts (e.g., demand-side management). Further changes are inevitable to facilitate the energy transition and to achieve corresponding ambitious economic, ecological, and political goals.

However, these changes may affect a variety of stakeholders in the grid, such as individual people, enterprises, cities, and whole countries. All stakeholders can, to some degree, affect upcoming changes in the energy grid. For instance, people can vote to influence politics. Enterprises and cities can, similarly, influence technical advancements or regulatory frameworks to support or impede changes. Increased knowledge about the energy grid can support upcoming changes in various ways: for individual people, knowledge can improve their affinity for energy-related topics, and allow them to improve their assessment of upcoming changes.^47^ Therefore, such knowledge can support their decision making toward investing into their own transition from consumers to prosumers. Moreover, people may become increasingly open-minded toward energy-related change, potentially speeding-up political decisions and technological transfer.

### Improving energy awareness

In the current energy grid, consumers are able to freely use electricity; simultaneously, the adoption of programs to improve the local energy management (e.g., to reduce peak loads and optimize the electricity consumption) is limited and their success is low.^1^^,^^34^ Increasing the understanding of the importance of ecological energy management, and fostering the motivation of people to be increasingly involved in processes that support this goal, may have a strong impact on both the adaption and the success of the aforementioned programs. In addition, such an improved awareness for energy-related topics may support the development and integration of novel programs. For instance, processes for integrating privately owned resources into resilient grid operation (e.g., demand-side management) may be simplified, e.g., by enabling prosumers to offer their EV as a battery storage unit that provides regulatory capacity for frequency control operations.

### Increasing preparedness and improving local decision making

Resilience is concerned with a system’s capability to withstand and absorb events that disrupt its stable operation.^48^ SGs are envisioned to strongly integrate consumers and prosumers alike, which are actively contributing to maintaining the stable operation of SG. This strong integration means that aspects that may affect people’s capabilities to fulfill their role in such a system may have a direct impact on the overall system stability. Consequently, the ability of people to deal with problematic events directly affects the resilient operation of the energy grid. In this context, we highlight two important aspects: people’s ability to estimate the *severity* of a problem situation (i.e., how strongly people are affected by the problem situation and the potential consequences); and the ability of people to *mitigate* the impact of the problem situation on them and on their environment (e.g., conducting actions to improve the problem situation). In the following, these aspects are explained in more detail:

• *Severity.* Increasing the energy-related knowledge of people can improve their ability to assess the impact of upcoming and ongoing problem situations.^47^ This understanding allows them to better compare current situations to previously experienced events and draw conclusions, becoming more resilient in the process, and improving the handling of future problems. For instance, understanding the fundamental concepts of blackouts, their main causes, and the established operational routines of energy grids for restarting impacted areas can support people in their local decision making during such an event. As a consequence, people may remain increasingly calm during blackout situations, since they know what local actions to conduct and which processes are set into motion by responsible authorities to resolve the situation. This can prevent actions that may impede the quick mitigation of the problem (e.g., activating many devices repeatedly) and, therefore, both supports the situation for individuals and alleviates the burden for control entities.
• *Mitigation.* Problem mitigation is a process that starts with preemptive measures for increasing the ability of an individual to deal with upcoming problems. People with increased knowledge about resilient grid operation may perceive personal preparations for handling emergency situations as more important compared with people who lack knowledge in this area (e.g., people may maintain local storage of water, food, and medical supply). As a consequence, a growing number of well-prepared people can alleviate the burden for personnel responsible for mitigating the problem situation. More precisely, since people may feel a greater sense of security—as their essential needs are covered—they can handle the situation more calmly. For instance, during large-scale problem situations, such as a blackout, stress on emergency hotlines is increasing significantly, which can lead to a hotline overload.^49^ Among these calls are a large number of cases that do not require an immediate response but are of lower severity. As a consequence, several high-severity calls may not reach the emergency operators, which can ultimately lead to casualties. Therefore, the increasingly calm behavior of people can reduce the stress on both technical and emergency hotlines due to a reduction in unnecessary calls. Additional benefits become apparent considering people’s role as prosumers in SGs. With the adoption of novel structural concepts, such as holarchies^50^ and the accompanying growing impact of individuals on maintaining the stable operation of the grid, the importance of mitigating problems locally is intensified. People who understand the significance of their role in such a context and are increasingly aware of their responsibilities and impact, can support the SG by conducting local decisions that support the mitigation of the problem situation.^1^ For instance, people may be able to offer additional local resources to be used in the mitigation process (e.g., EVs using vehicle-to-grid technology). Such actions can directly support the mitigation of the problem at hand. In addition, informed and aware people may decide to use locally available alternatives instead of electrically powered appliances (e.g., alternative ways of heating) since they know that this reduction in electricity consumption may help to supply more important appliances in the grid. However, such decision making is only feasible if people are capable of understanding the problem situation; therefore, they need profound knowledge about available actions and measures that can be taken to improve the situation locally.

## Conclusion

The energy grid is a complex socio-technical system that is undergoing drastic changes. While the majority of the emerging technical challenges are well understood, the challenges affecting the social components in the grid are often not considered. In this work, we highlight the importance of the involvement of people for the resilient operation of future energy grids. We investigate the reasons for the dominantly passive role of consumers in current energy grid operations and elaborate on the high degree of energy grid availability as one of the main reasons. We then explore how energy grid data, increased domain knowledge, and improved motivation for participation can affect the resilient operation of future energy grids, and present schemes that can support the involvement of people. Our ongoing and future research efforts aim at investigating the willingness for participation of people empirically and exploring technical concepts that leverage various types of data for empowering people to contribute to the resilience of future energy grids.
